# Efficacy of robotic radical hysterectomy for cervical cancer compared with that of open and laparoscopic surgery: A separate meta-analysis of high-quality studies

**DOI:** 10.1097/MD.0000000000014171

**Published:** 2019-01-25

**Authors:** Sha-sha Zhang, Tian Ding, Zheng-hui Cui, Yuan Lv, Ruo-an Jiang

**Affiliations:** Department of Obstetrics and Gynecology, Women's Hospital, Zhejiang University of Medicine, Hangzhou, Zhejiang Province, China.

**Keywords:** cervical cancer, hysterectomy, laparoscopy, meta-analysis, robot

## Abstract

**Background:**

To perform a meta-analysis of high-quality studies comparing robotic radical hysterectomy (RRH) vs laparoscopic radical hysterectomy (LRH), and open radical hysterectomy (ORH) for the treatment of cervical cancer.

**Methods:**

A systematic search of PubMed, Embase, Cochrane Library, and Web of Science was performed to identify studies that compared RRH with LRH or ORH. The selection of high-quality, nonrandomized comparative studies was based on a validated tool (methodologic index for nonrandomized studies) since no randomized controlled trials have been published. Outcomes of interest included conversion rate, operation time, intraoperative estimated blood loss (EBL), length of hospital stay (LOS), morbidity, mortality, number of retrieved lymph nodes (RLNs), and long-term oncologic outcomes.

**Results:**

Twelve studies assessing RRH vs LRH or ORH were included for this meta-analysis. In comparison with LRH, there was no difference in operation time, EBL, conversion rate, intraoperative or postoperative complications, LOS, and tumor recurrence (*P* > .05). Compared with ORH, patients underwent RRH had less EBL (weighted mean difference [WMD] = −322.59 mL; 95% confidence interval [CI]: −502.75 to −142.43, *P* < .01), a lower transfusion rate (odds ratio [OR] = 0.14, 95% CI: 0.06–0.34, *P* < .01), and shorter LOS (WMD = −2.71 days; 95% CI: −3.74 to −1.68, *P* < .01). There was no significant difference between RRH and LRH with respect to the operation time, intraoperative or postoperative complications, RLN, and tumor recurrence (*P* > .05).

**Conclusion:**

Our results indicate that RRH is safe and effective compared to its laparoscopic and open counterpart and provides favorable outcomes in postoperative recovery.

## 1 Introduction

Despite the fact that the Pap smear has become widely available, there is increasing use of human papilloma virus tests and vaccination, and the incidence of cervical cancer has decreased,^[1]^ cervical cancer still remains the 2nd most common cause of cancer death for women, especially in developing countries.^[2]^ The principle treatment option to improve the survival rate is still surgical resection with adequate lymphadenectomy. Abdominal radical hysterectomy has been traditionally considered the standard of care for women with resectable cervical cancer. In 1992, Nezhat et al 1st reported the use of laparoscopic radical hysterectomy (LRH) to treat cervical cancer.^[3]^ Since then, LRH has prevailed in producing satisfactory surgical outcomes over the conventional vaginal approach.^[4]^ However, LRH has some inherent drawbacks. First, a flat, 2-dimensional image, and the reduced tactile feedback in LRH demand that surgeons have fine hand-eye coordination. Second, the limited motion of the nonarticulating laparoscopic instruments leads surgeons to operate in an awkward and uncomfortable position. Though not clearly defined, these drawbacks are associated with a long learning curve time and an exhausting surgical experience. Robotic surgical techniques have been reported to be more favorable in abdominal surgery.^[5,6]^ An increasing number of studies have reported the benefits of robotic radical hysterectomy (RRH) including better ergonomics, higher definition, the ability to see in 3-dimensionals, 7° of wrist-like motion, tremor filtering, motion scaling, and less fatigue.^[7]^

However, RRH has not been sufficiently studied in well-designed prospective randomized trials (RCTs). The meta-analyses available for RRH have therefore included the available nonrandomized comparative studies (NRCTs) to overcome the paucity of RCTs.^[8,9]^ Thus unreliable results and little strong evidence had been presented. On the contrary, there is evidence that estimates derived from high-quality NRCTs may be similar to those derived from RCTs.^[10]^ Also, when comparing surgical procedures, the pooling of high-quality NRCTs could be as accurate as pooling RCTs.^[11]^ Accordingly, we have conducted this updated meta-analysis of RRH comparing it with LRH and ORH for the treatment of cervical cancer.

## 2 Materials and methods

### 2.1 Systematic literature search

Systematic searches of PubMed, Embase, Cochrane Library, and Web of Science were performed to identify articles published up to February 2018. Search strategies using the logical combinations of keywords are as follows: “minimally invasive,” “robot,” “robotic,” “Da Vinci,” “hysterectomy,” and “cervical cancer.” All eligible studies in English were retrieved, and their “relevant articles” and bibliographies were checked for potential relevant publications.

### 2.2 Eligibility criteria

The inclusion criteria for systematic review and meta-analysis were prospective or retrospective cohorts assessing surgical outcomes of RRH; comparing interested surgical outcomes of RRH with LRH or ORH. The following studies or data were excluded if they met the following criteria:

1. Case reports, reviews, letters, editorials, and studies lacking control groups were excluded.
2. Studies reported on a comparison of RRH vs LRH or ORH for patients with benign lesions or gynecologic malignancy other than cervical cancer such as endometrial cancer.
3. Overlapped studies.
4. Impossible to extract any of the interested outcomes.

Then, the methodologic quality of the eligible NRCTs was assessed by the methodologic index for nonrandomized studies (MINORS).^[12]^ In total, 8 items were evaluated, with a maximum score of 16 points. Studies with 12 or more points were considered as high quality and were included in the meta-analysis. Those with <12 points were excluded.

### 2.3 Data extraction and quality assessment

Two investigators (ZSS and DT) independently assessed publications for inclusion in the article. Discrepancies between the 2 reviewers were resolved via discussion with a 3rd senior author (JRA). Data extracted from eligible studies included the baseline characteristics, such as 1st author, publication period, region, study type, sample size, the International Federation of Gynecology and Obstetrics stage. Interested outcomes were extracted and compared including conversion rate, operation time, intraoperative estimated blood loss (EBL), length of hospital stay (LOS), morbidity, mortality, number of retrieved lymph nodes (RLN), margin distance, and long-term oncologic outcomes. The Newcastle–Ottawa Quality Assessment Scale was utilized to evaluate the quality of the studies included (http://www.ohri.ca/programs/clinical_epidemiology/oxford.asp).

### 2.4 Statistical analysis

For the comparison analysis of dichotomous variables (e.g., postoperative morbidities) among surgical methods, we employed the odds ratio (OR) with 95% confidence interval (CI). Weighted mean difference (WMD) with 95% CI was used for continuous parameters (e.g., operation time and blood loss). The means and standard deviations (SDs) were estimated as described by Hozo et al^[13]^ if the research offered medians and ranges rather than means and SDs. Statistical heterogeneity, which indicated between-study variance, was evaluated according to the Higgins *I*^2^ statistic.^[14]^ Based on DerSimonian and Laird's approach, the random-effects model was utilized to account for clinical heterogeneity that means diversity in a sense which is related to clinical situations. According to the general complication, the bias of potential publication was determined by carrying out informal visual inspection of funnel plots. A 2-tailed value of *P* < .05 was considered significant. All statistical tests were performed with Review Manager Version 5.1 (The Cochrane Collaboration, Oxford, England).

## 3 Results

### 3.1 Study eligibility and characteristics

**Figure 1:**
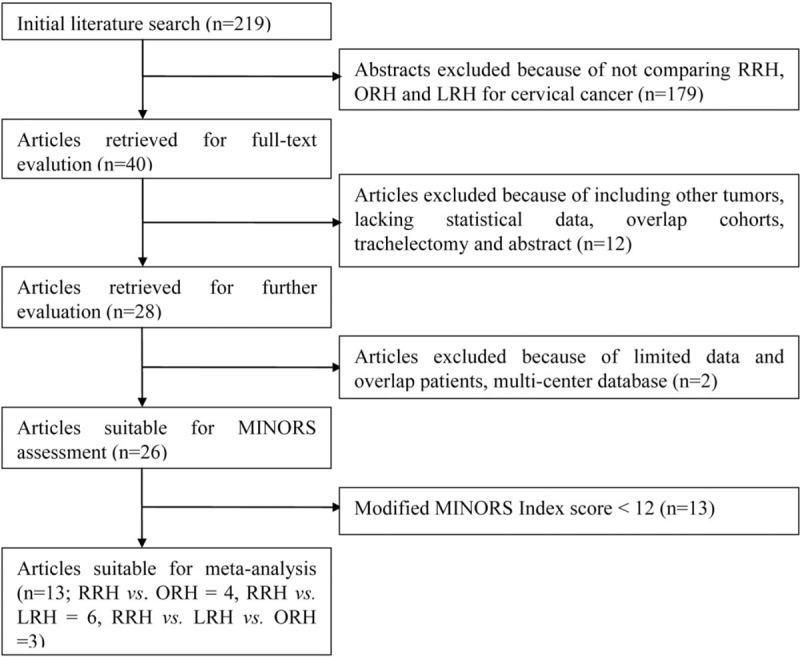
Flow chart of literature search strategies.

Initially 219 articles were identified for further selection from the medical databases. Next, 164 articles were excluded after title screening or abstract screening. Continually, a further 12 studies were excluded when full text was read due to including tumors other than cervical cancer (n = 4), lacking statistical data (n = 3), overlap patient cohorts (n = 4), and trachelectomy instead of hysterectomy (n = 1). Then 26 studies were selected for quality assessment, 13 studies were excluded by a modified MINORS index score <12.^[15–27]^ Finally, 13 studies were selected for further meta-analysis.^[28–40]^ The MINORS assessment of these studies is showed in Table 1. A flow chart of the search strategies, which contains reasons of excluded studies, is elucidated in Figure 1. A total of 2197 patients were included in the analysis with 932 undergoing RRH, 373 undergoing LRH, and 892 undergoing ORH. They represented an international experience (3 the United States, 2 South Korea, 4 Italy, 1 France, 1 Sweden, one 2-center study of the United States and Italy, one 3-center study of states United States and Norway). Four studies compared RRH to ORH, 6 compared RRH and LRH, and remaining 3 studies provided comparative data for RRH, LRH, and ORH within the same study. Table 2 lists the studies identified and their main characteristics.

**Table 1:**
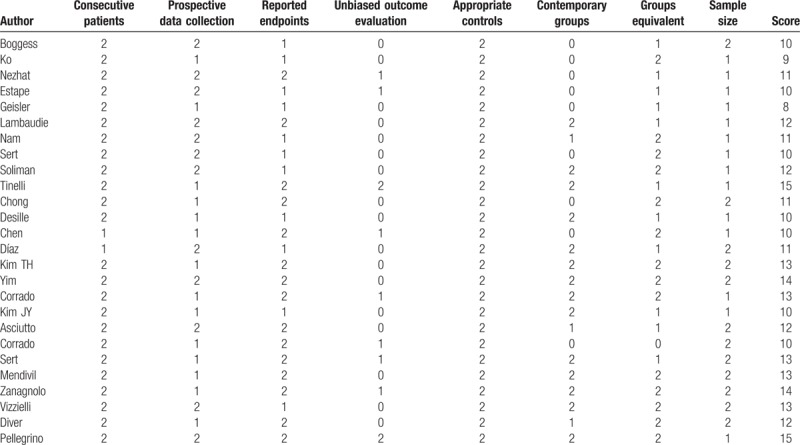
Modified MINORS score of the studies.

**Table 2:**
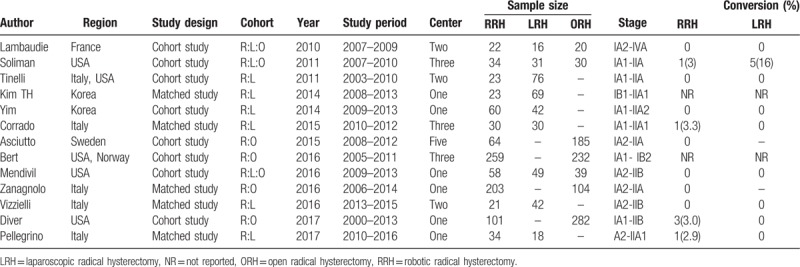
Summary of studies included in the meta-analysis.

### 3.2 Short-term outcomes

#### 3.2.1 Robotic radical hysterectomy vs laparoscopic radical hysterectomy

**Figure 2:**
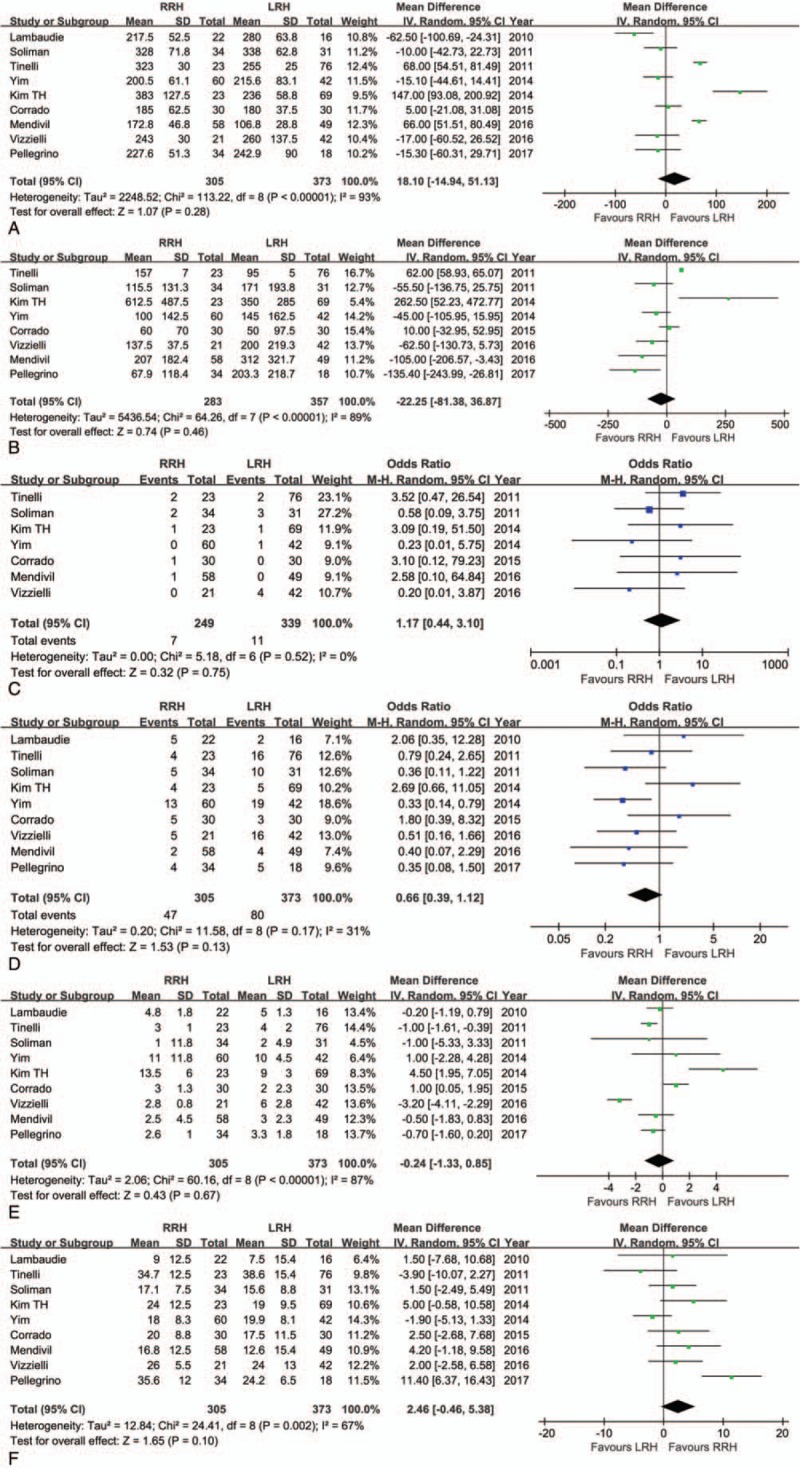
Forest plot of the meta-analysis: robotic radical hysterectomy (RRH) vs laparoscopic radical hysterectomy (LRH). (A) Operation time. (B) Estimated blood loss. (C) Intraoperative complications. (D) Postoperative complications. (E) Length of hospital stay. (F) Retrieved lymph nodes.

There was no significant difference in operation time between the 2 groups (WMD = 18.10 minutes; 95% CI: −14.94 to 51.13, *P* = .28) (Fig. 2A). Both as minimally invasive surgery, RRH did not showed a priority in EBL (WMD = −22.25 mL; 95% CI, -81.38–36.87, *P* = .46) (Fig. 2B) and transfusion rate (OR = 0.53; 95% CI: 0.16–1.75, *P* = .29). The conversion to open surgery rate was similar between RRH and LRH (OR = 0.66; 95% CI: 0.09–4.67, *P* = .68). Pooled data showed that there was no significant difference in intraoperative mortality (OR = 1.17; 95% CI: 0.44 between RRH and LRH 3.10, *P* = .32) (Fig. 2C) or postoperative morbidity (OR = 0.66; 95% CI: 0.39–1.12, *P* = .13) (Fig. 2D). With respect to LOS, there was also no significant difference between RRH and LRH (WMD = −0.24 days; 95% CI: −1.33 to 0.85, *P* = .67) (Fig. 2E). The mean number of RLN was larger for robotic surgery than for laparoscopic procedure (WMD = 2.46; 95% CI: −0.46 to 5.38, *P* = .10) (Fig. 2F). However, the surgical margins such as the right and left parametrium, as well as the vaginal edge could not be determined due to limited data. The meta-analysis for RRH and LRH are outlined in Table 3.

**Table 3:**
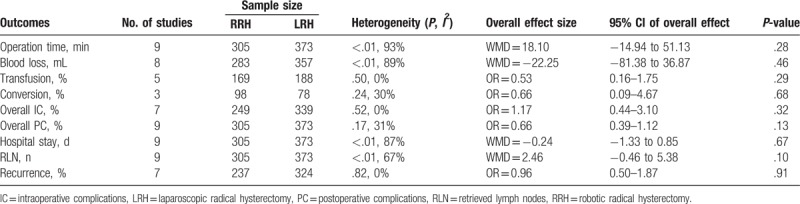
Meta-analyses results for robotic radical hysterectomy vs laparoscopic radical hysterectomy.

#### 3.2.2 Robotic radical hysterectomy vs open radical hysterectomy

**Figure 3:**
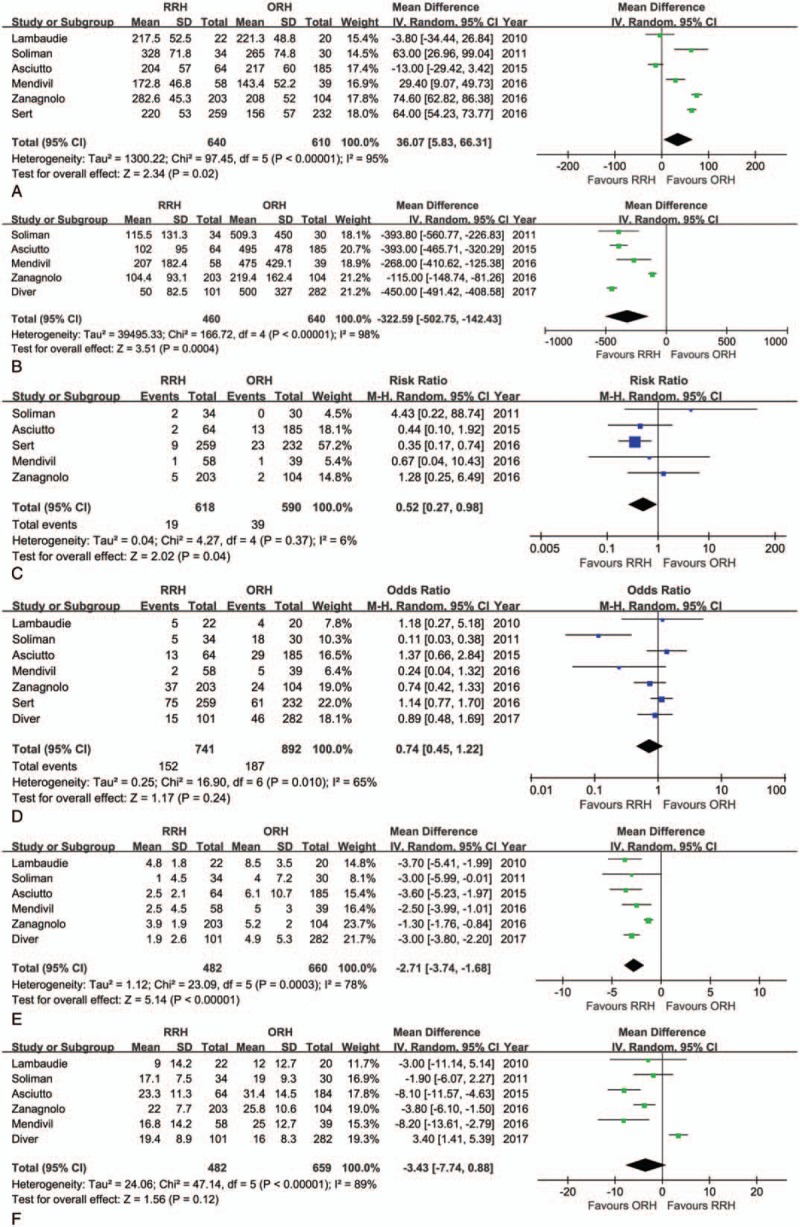
Forest plot of the meta-analysis: robotic radical hysterectomy (RRH) vs open radical hysterectomy (ORH). (A) Operation time. (B) Estimated blood loss. (C) Intraoperative complications. (D) Postoperative complications. (E) Length of hospital stay. (F) Retrieved lymph nodes.

The mean operation time was shorter for ORH than for RRH (WMD = 36.07 minutes; 95% CI: 5.83–66.31, *P* = .02) (Fig. 3A), however RRH significantly reduced the EBL (WMD = −322.59 mL; 95% CI: −502.75 to 142.43, *P* < .01) (Fig. 3B) and the transfusion rate (OR = 0.19, 95% CI: 0.09–0.39, *P* < .01). There were less overall intraoperative complications in RRH than ORH (OR = 0.52, 95% CI: 0.27–0.98, *P* = .04) (Fig. 3C), but there were no significant differences in the rate of postoperative complications seen between the RRH and ORH groups (OR = 0.74, 95% CI: 0.45–1.22, *P* = .24) (Fig. 3D). A shorter LOS was also observed in the RRH group (WMD = −2.71 days; 95% CI: −3.74 to −1.68, *P* < .01) (Fig. 3E). The difference in RLN between RRH and ORH was not statistically significant (WMD = −3.43; 95% CI: −7.74 to 0.88, *P* = .12) (Fig. 3F). The meta-analysis results for RRH and ORH are shown in Table 4.

**Table 4:**
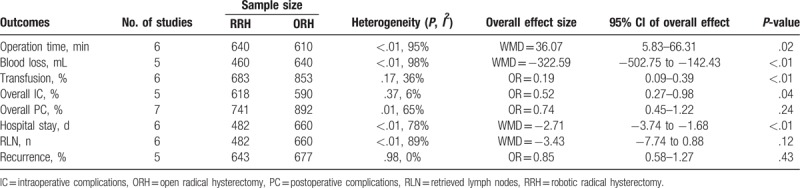
Meta-analyses results for robotic radical hysterectomy vs open radical hysterectomy.

#### 3.2.3 Long-term outcomes

**Figure 4:**
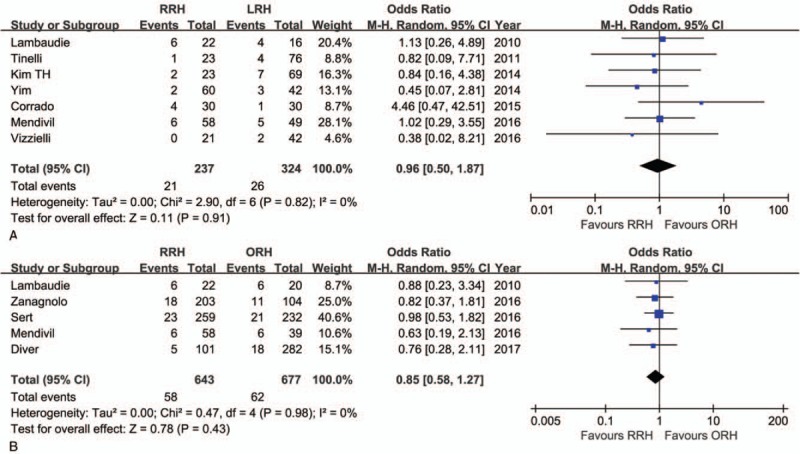
Forest plot of the meta-analysis for recurrence. (A) Robotic radical hysterectomy (RRH) vs laparoscopic radical hysterectomy (LRH). (B) RRH vs open radical hysterectomy (ORH).

A systematic review of outcomes including follow-up time, recurrence, and long-term survival rates are summarized in Table 5. The pooled data indicate that there were no significant differences between RRH and LRH (OR = 0.96; 95% CI: 0.50–1.87, *P* = .91) (Table 3, Fig. 4A). Furthermore, with respect to recurrence, the difference between RRH and ORH was also not significant (OR = 0.85; 95% CI: 0.58–1.27, *P* = .43) (Table 4, Fig. 4B). Long-term survival rates were reported in 8 studies, and there were no considerable differences in survival rates between the RRH group and the ORH group or between the RRH group and the LRH group.^[28,30–32,35,37,39,40]^ Unfortunately, a meta-analysis of survival rates could not be performed due to the limited data.

**Table 5:**
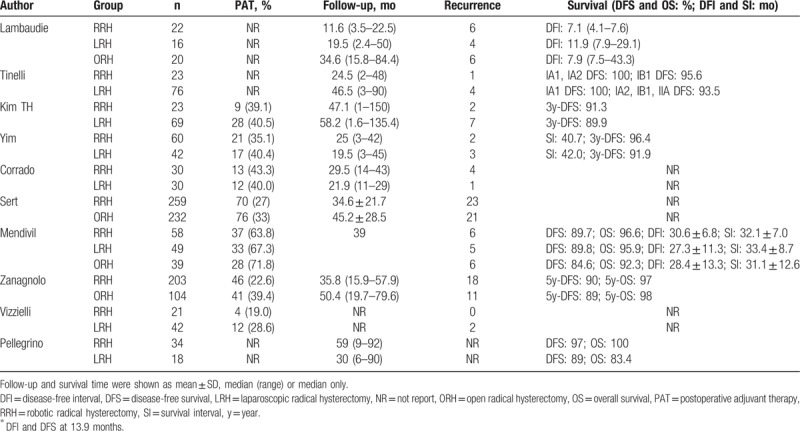
Systematic review of recurrence and long-term survivals.

#### 3.2.4 Publication bias

**Figure 5:**
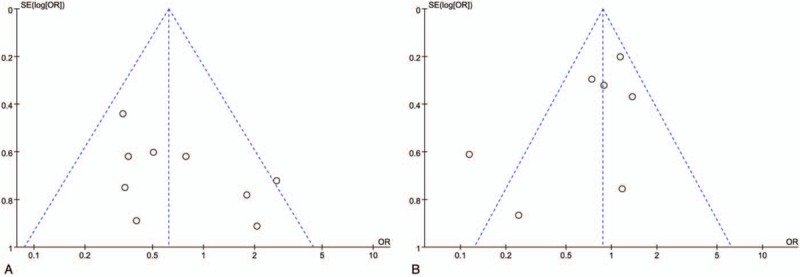
Funnel plots of the overall postoperative complications rates. (A) Robotic radical hysterectomy (RRH) vs laparoscopic radical hysterectomy (LRH). (B) RRH vs open radical hysterectomy (ORH).

The funnel plot for studies reporting the RRs of postoperative morbidity was used to detect the publication bias. For RRH vs LRH, the study by Kim et al was partly outside the funnel.^[31]^ As to RRH vs ORH, the study by Soliman et al was partly outside the funnel,^[29]^ whereas the remaining representative plots were distributed symmetrically. We believed such publication bias was acceptable in the studies (Fig. 5).

## 4 Discussion

Although recent publications have questioned the use of radical surgery for small tumors, the standard of care for surgical therapy remains radical hysterectomy for cervical cancer, regardless of histology.^[41]^ Innovations continue to improve surgical outcomes, lighten surgeons’ workload, and improve patients’ experience. Minimally invasive surgery is one of the most outstanding innovations in recent decades. LRH, a well-known minimally invasive approach for the treatment of cervical cancer, has been widely adopted because of the benefit it provides, although it has some deficiencies. Robotic surgery was introduced with the expectation that it would prove to be optimal and would displace laparoscopic surgery as the conventional approach.^[5]^ In the cervical cancer field, RRH efforts have been ongoing since 1st described by Sert and Abeler in 2006.^[42]^

In general, an innovation in surgery should bring about a significant benefit for the patients or surgeons before it becomes widely used. The benefits should be well-defined and the appraisal tools should be comparable, reproducible, and convincing. This meta-analysis selected and summarized high-quality literature that compared the short- and long-term outcomes in the treatment of cervical cancer. We believe such a meta-analysis contributes a more objective and comprehensive assessment for the current RRH surgical status in cervical cancer.

Our analyses highlighted an advantage of RRH in minimal surgical trauma since less intraoperative blood loss and shorter postoperative hospital stays were observed. In contrast, the operation time was similar between groups. Further analyses of intraoperative or postoperative morbidity, lymph node harvest, and margin status between RRH and ORH did not show any significant differences. Comparing RRH and LRH, we found they were similar in operation time, blood loss, intraoperative or postoperative morbidity, and the length of hospital stay. Similar to most reports comparing robotic and open surgery in many different clinical situations,^[6]^ intraoperative bleeding in the RRH group was less than that in the ORH group, as well as the need for transfusions. The meticulous dissection of tissues and vessels due to the superior vision available in RRH, the application of energy-dividing devices and the reduced length of the surgical incision wound all contribute to this reduction in blood loss. The most striking finding was a similar operation time between RRH and, or ORH, whereas in many other different abdominal operations, a longer operation time is usually found for robotic approaches.^[5]^ This discrepancy might arise from the fact RRH requires only resection, not reconstruction. RRH may need long time for docking before operation in unexperienced hands. Thus, we inferred the “actual time” for surgeons performing RRH might be shorter than that for LRH and ORH. Radical hysterectomy is usually performed with both limited surgical place and vision. RRH provide improved ergonomics, better anatomical insight, and more flexible jaws instead of hands that all contribute to shortening the operation time, alleviating surgeons fatigue and optimizing surgical outcomes. Many surgeons may have considerable experience with LRH before performing RRH, which helps them adapt quickly to the robotic procedure. In addition, the enhancements of robotic surgery, such as wristed instrumentation and improved vision, also allow for surgeons to quickly master the robotic technique. Therefore, the learning curve for performing RRH does not necessarily have to be long.

With respect to oncologic outcomes, we found that the mean number of RLNs had a tendency to be higher in RRH than for LRH, even though many RRH cases were 1st cases. Lymph node metastasis of cervical cancer often signifies an aggressive or advanced disease. Adequate lymphadenectomy is crucial elements of radical hysterectomy and for achieving better oncologic outcomes. The EndoWrist function allows the surgeon to reach in-depth field which would be unavailable with regular straight forceps utilized in the regular laparoscopic surgery thus contributing to the favorable result.^[43]^ Therefore, we believe that robotic surgery could be superior to the conventional laparoscopic procedure in lymphadenectomy. Although the margin status could not be evaluated due to the limited data, the evidence available suggests that RRH is not inferior.^[21,27,29,34,36]^ Recurrence and long-term survival rates are direct outcomes for evaluating the oncologic efficacy of RRH. Based on the data available, postoperative cancer recurrence and long-term survival rates for RRH were not inferior to those in ORH. In addition, in the study of Ko et al,^[16]^ patients received radiation or radiochemotherapy postoperatively, and all patient survived during the limited follow-up time. Nezhat et al^[17]^ reported no recurrence in either group, with a mean follow-up time of 12 months in the RRH group and 29 months in the LRH group. Díaz-Feijoo et al^[25]^ reported 1 node recurrence in a patient received a 2nd robotic-assisted transperitoneal node debulking, and 3 patients of progression, all of whom died of their disease. However, as the majority of cases in the present study were early cervical cancer, the effect of RRH for treating early cervical cancer is promising. However, there should be a note of caution for using RRH in advanced cervical cancer because the relevant studies and clinical evidence are still lacking.

Some of our research limitations are as follows:

1. No RCTs were included: Due to the higher cost of robotic surgery, no RCTs on RRH have yet been conducted. A sequence of biases is therefore produced because the trial designs used are not RCTs, and these biases present in our study limit the conclusions we can reach. There is a currently an ongoing international phase III RCT underway comparing laparoscopic or RRH to abdominal radical hysterectomy for early cervical cancer that initiated recruitment in 2008.^[44]^ Target enrollment is 740 patients and is estimated to reach accrual in 2018.
2. Heterogeneities amongst the included studies, especially comparing the operation time, intraoperative blood loss, and length of hospital stay, would also result in bias. These parameters are influenced by the heterogeneities in the surgeon's surgical skills, patients’ conditions, and the perioperative care protocol.
3. The long-term survival rate was not calculated because of limited data, and this may affect our confidence of outcomes.

## 5 Conclusion

The RRH is safe, effective, and comparable to ORH and LRH, or even more favorable with respect to outcomes in surgical trauma and postoperative recovery. Further prospective, multicentric and large sample randomized control trials are needed to confirm our findings. We expected that a phase III randomized trial by Obermair et al^[44]^ will offer more definitive answers.

## Author contributions

**Conceptualization:** Sha-sha Zhang.

**Data curation:** Sha-sha Zhang.

**Formal analysis:** Tian Ding.

**Investigation:** Yuan Lv.

**Methodology:** Tian Ding, Yuan Lv.

**Resources:** Zheng-hui Cui.

**Supervision:** Ruo-an Jiang.

**Writing – original draft:** Sha-sha Zhang.

**Writing – review & editing:** Ruo-an Jiang.
